# Efficacy and Feasibility of Remote Cognitive Remediation Therapy in Parkinson's Disease: A Randomized Controlled Trial

**DOI:** 10.1155/2023/6645554

**Published:** 2023-06-01

**Authors:** Lisa Hoffman, Nicholas D. Burt, Nicholas R. Piniella, Madison Baker, Nicole Volino, Saeed Yasin, Min-Kyung Jung, Adena Leder, Amber Sousa

**Affiliations:** ^1^Department of Family Medicine, New York Institute of Technology College of Osteopathic Medicine, Old Westbury, NY, USA; ^2^New York Institute of Technology College of Osteopathic Medicine, Old Westbury, NY, USA; ^3^Department of Research, New York Institute of Technology College of Osteopathic Medicine, Old Westbury, NY, USA; ^4^Department of Osteopathic Manipulative Medicine, New York Institute of Technology College of Osteopathic Medicine, Old Westbury, NY, USA

## Abstract

**Background:**

Non-motor symptoms of Parkinson's disease (PD) such as cognitive impairment are common and decrease patient quality of life and daily functioning. While no pharmacological treatments have effectively alleviated these symptoms to date, non-pharmacological approaches such as cognitive remediation therapy (CRT) and physical exercise have both been shown to improve cognitive function and quality of life in people with PD.

**Objective:**

This study aims to determine the feasibility and impact of remote CRT on cognitive function and quality of life in patients with PD participating in an organized group exercise program.

**Methods:**

Twenty-four subjects with PD recruited from Rock Steady Boxing (RSB), a non-contact group exercise program, were evaluated using standard neuropsychological and quality of life measures and randomized to the control or intervention group. The intervention group attended online CRT sessions for one hour, twice a week for 10 weeks, engaging in multi-domain cognitive exercises and group discussion.

**Results:**

Twenty-one subjects completed the study and were reevaluated. Comparing groups over time, the control group (*n* = 10) saw a decline in overall cognitive performance that trended towards significance (*p* = 0.05) and a statistically significant decrease in delayed memory (*p* = 0.010) and self-reported cognition (*p* = 0.011). Neither of these findings were seen in the intervention group (*n* = 11), which overwhelmingly enjoyed the CRT sessions and attested to subjective improvements in their daily lives.

**Conclusions:**

This randomized controlled pilot study suggests that remote CRT for PD patients is feasible, enjoyable, and may help slow the progression of cognitive decline. Further trials are warranted to determine the longitudinal effects of such a program.

## 1. Introduction

Although the most prominent symptoms of Parkinson's disease (PD) are motor in nature, non-motor symptoms such as cognitive impairment are common [1]. Cognitive deficits in the domains of attention, memory, executive function, and visuospatial construction can decrease patient quality of life and overall functioning and, when combined with the progression of PD, can lead to mild cognitive impairment (MCI) and even dementia [1–4]. Several mechanisms are thought to contribute to this cognitive decline, including protein misfolding, synaptic pathology, neurotransmitter activity, mitochondrial dysfunction, and neuroinflammation [2]. However, no pharmacological interventions have been effective in alleviating cognitive impairment in PD to date [5].

Cognitive remediation therapy (CRT), sometimes referred to as cognitive training or cognitive rehabilitation, is a neuropsychological treatment consisting of exercises targeting specific thinking skills with the goal of improving overall cognitive functioning. CRT has been demonstrated to be effective in maintaining or improving cognitive health not only in healthy populations [6, 7] but also in many disease groups including Alzheimer's disease [8], multiple sclerosis [9], schizophrenia [10], and PD [4, 11–13]. Meta-analyses of cognitive training trials with PD patients have indicated improved performance specifically in memory, executive function, and processing speed [4, 12]. In one such study, participation in the program was even associated with increased brain connectivity and activation, as determined by fMRI [14]. In addition to these benefits, CRT can also be made accessible via remote, online administration.

A systematic review of 24 remote cognitive remediation studies demonstrated that home-based cognitive training programs are not only feasible but also well-tolerated and useful for many different disease groups [15]. One of these studies, a single-arm trial for PD patients, claimed improvements not only in global cognitive scores but also in some aspects of mobility, specifically on the Timed Up and Go (TUG) test [16]. A more recent review found that there was no significant difference in the efficacy of remote computerized cognitive training for PD patients compared to in-person programs [17]. Of the five home-based programs included, however, the majority lasted five weeks or less, trained in just one cognitive domain, or only included patients without cognitive impairment [18–22].

Physical exercise has likewise been demonstrated to maintain or improve cognitive function and quality of life in patients with PD [23, 24], and it has been suggested that a combination with CRT might augment the benefits of both programs [11]. One such study combined a multi-domain cognitive intervention with motor rehabilitation and saw improvements in cognitive performance compared to motor rehabilitation alone [25], while another study found that cognitive and psychomotor training were superior to cognitive training alone [26]. It is evident, however, that the combination of these non-pharmacological approaches has yet to be fully explored. Therefore, this study aims to determine the feasibility of a remote CRT program for PD patients and its impact on cognition, functioning, and quality of life.

## 2. Methods

### 2.1. Study Design

This was a randomized, controlled, non-blinded pilot study designed to evaluate the impact of remote CRT on cognition and quality of life in PD patients recruited from a boxing exercise program. Participants were randomized into either an intervention or control group. All subjects underwent neuropsychological evaluation using the Montreal Cognitive Assessment (MoCA) and Repeatable Battery for the Assessment of Neuropsychological Status (RBANS) at baseline and following the intervention. The trial was approved by the New York Institute of Technology (NYIT) Institutional Review Board (IRB) and registered with clinicaltrials.gov (NCT04955275).

### 2.2. Power Analysis

A power analysis was performed based on the reported values for RBANS total scores for a healthy population (minimum clinically important difference (MCID) of 8 points and a mean of 100 with a standard deviation of 15) [27]. The expected effect size was estimated to be medium (*d* = 0.5). Following the guidelines on sample size for pilot studies, it was determined that recruiting 12 participants per arm, with an anticipated 15% attrition rate, would be adequate to assess both feasibility and efficacy while achieving 80% statistical power [28].

### 2.3. Subjects

Inclusion criteria for this study were as follows: Diagnosis of PD, enrollment in Rock Steady Boxing (RSB; an in-person non-contact group exercise program designed specifically for PD patients) at the NYIT College of Osteopathic Medicine (NYITCOM), age between 40 and 85 years old, MoCA score of 20 or above, and capable of utilizing a computer and mouse. Exclusion criteria were as follows: Participation in concurrent research study or other interventional clinical trial 30 days prior to consenting and history of intellectual disability or other comorbid neurological disorder.

Twenty-five individuals with PD were recruited from the NYITCOM RSB program. All 25 individuals signed an IRB-approved consent form and underwent neuropsychological evaluation. Of those, 24 subjects (96.0%) met inclusion criteria and went on to participate in the study. All subjects were instructed to continue attending RSB at least once per week in order to remain enrolled in the study.

### 2.4. Assessment of Cognition and Quality of Life

Neuropsychological and cognitive functions were evaluated using MoCA and RBANS. These assessments were selected to measure performance in the cognitive domains of attention, memory, executive function, visuospatial construction, semantic fluency, problem-solving, and psychomotor speed. To assess quality of life, the Parkinson's Disease Questionnaire (PDQ-39) was also administered. All evaluations were administered by medical students trained by a licensed neuropsychologist. Each subject was assessed within six weeks prior to the start of the intervention and reassessed within three weeks after the intervention ended. RBANS forms A and B were counterbalanced across time points. Select quotes regarding the CRT program were transcribed during the discussion periods and systematically categorized into domains based on the PDQ-39 and themes derived from RBANS subsections. A custom, online, eight-question Likert scale questionnaire designed to assess subjective improvements seen in subjects' daily lives was also administered following the final CRT session, prior to follow-up evaluation.

### 2.5. Randomization

Subjects were sequentially de-identified, and stratified randomization was performed to assign subjects to the intervention or control group, such that the two groups were balanced for RSB class level (high-, medium-, or low-intensity) and RBANS form administered at the baseline (A or B). One manual adjustment was made to balance for sex. Subjects were informed of their group assignments and, due to the size of the research team and nature of the intervention, post-intervention evaluators were not blinded to their assignments.

### 2.6. Intervention

BrainHQ (Posit Science Corporation, San Francisco, California) was utilized as the CRT intervention program for this study because of its evidence base consisting of hundreds of studies in various patient populations, including PD. BrainHQ is an adaptive computerized cognitive training program that is HIPAA compliant and tracks participant performance.

CRT sessions were held remotely via the online video conference platform Zoom (Zoom Video Communications, Inc., San Jose, California). Two introductory meetings were held prior to the start of the intervention sessions to familiarize subjects with the meeting platform and the CRT program. Sessions were held twice per week, for one hour, over 10 weeks. Sessions were facilitated by medical students and supervised by a licensed psychologist. Sessions consisted of 45 minutes of six exercises from BrainHQ, each targeting a specific cognitive domain. The exercises used in the study targeted attention (Double Decision), brain speed (Sound Sweeps and Fine Tuning), and memory (Memory Grid, Syllable Stacks, and To-Do List). At the conclusion of the exercise period, a 15-minute group discussion was held in order to discuss skills and strategies learned which can be generalized to everyday life tasks.

### 2.7. Analysis

The primary endpoint of this study was to assess changes in mean RBANS and PDQ-39 scores from baseline to post-intervention among groups. This analysis was limited to completers, those subjects who completed the primary endpoint measurements, and those who did not were excluded from the analysis. Outlier analysis was performed to identify and describe discreetly any abnormal observations using statistical procedures in which values were identified as extreme when greater than *Q*3 + 3 *∗* IQR or less than *Q*1 − 3 ∗ IQR. Baseline comparisons were made between the control and intervention groups to assess their equivalence and to detect any confounding variables. To describe the data, means and standard deviations were reported for the continuous variables and frequencies and percentages for the categorical variables. To compare between the intervention and control groups, the Mann–Whitney *U* test was used for the continuous variables and chi-square tests for the categorical variables. Simple pairwise comparisons between pre-intervention and post-intervention for the continuous outcomes were performed using the related-sample Wilcoxon signed rank test.

Generalized estimating equations (GEEs) were used to compare the multiple pairwise outcome data pre-intervention and post-intervention for the control and intervention groups. For the main outcomes of RBANS and PDQ-39, the total score and the subsection scores were compared pairwise pre-intervention and post-intervention within each group, control and intervention, and also between groups pre-intervention and post-intervention. The sequential Sidak method was used for multiplicity correction.

## 3. Results

### 3.1. Feasibility and Subjective Impact

**Figure 1:**
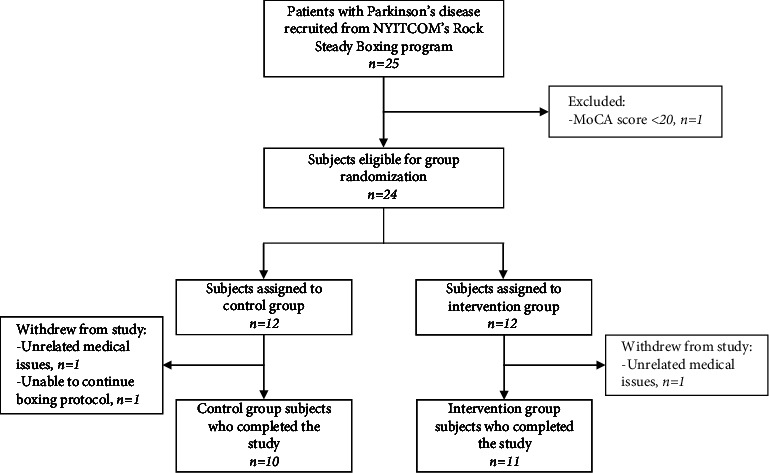
CONSORT flow chart of subject recruitment and participation.

Of the 24 randomized subjects, 21 subjects (68.7 ± 8.2 years old, 12 males), including 11 intervention and 10 control, completed the trial and follow-up evaluation. Three subjects (one intervention and two control; 12.5%) withdrew from the study due to unrelated medical issues (*n* = 2) and protocol noncompliance/nonadherence (*n* = 1) and were excluded from analysis (Figure 1). Subject demographics were similar between both groups (Table 1). Intervention adherence was high, with all 11 intervention subjects completing all 20 hours of BrainHQ training across the 10 weeks. There were 15 (7.6%) instances where a subject missed a live session and made up the cognitive exercise portion asynchronously.

**Figure 2:**
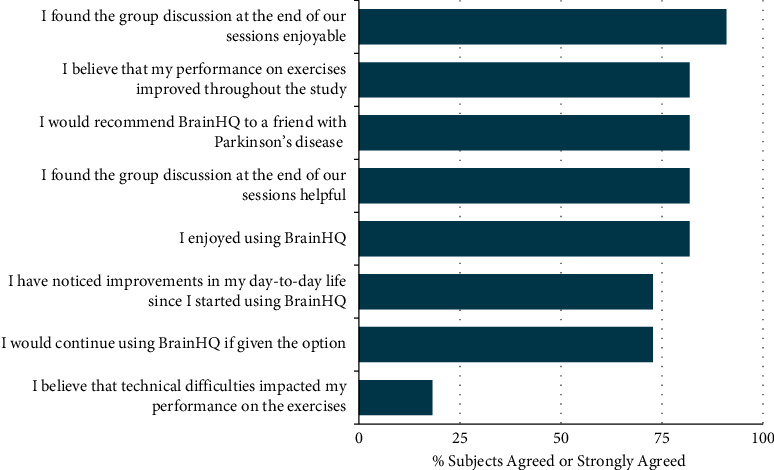
Results of the post-intervention Likert scale questionnaire. Reported as percent of the intervention group (*n* = 11) agreed or strongly agreed for each statement included.

Feedback given during the CRT sessions regarding the intervention and its impact was favorable, with subjects describing subjective improvements in memory, attention, self-confidence, and mood (Table 2). According to the survey given to intervention subjects following their last CRT session, subjects enjoyed both the group discussions (90.9%) and the cognitive exercises (81.8%) and noted improvements in their daily lives (72.7%) (Figure 2). Of the eight subjects who noticed improvements, they reported improved attention (100%), visuospatial perception (75%), memory (62.5%), and language ability (12.5%). Of the 11 intervention subjects, two (18.2%) believed that technical difficulties impacted their performance.

### 3.2. Objective Variable Analysis

Regarding analysis of the MoCA, RBANS, and PDQ-39 variables, our outlier analysis showed that none of the values were extreme, with just a few being moderate. With no significant outliers, all data points were included in the analysis. At baseline, the MoCA total scores (control: 23.9, intervention: 26.1, *p* = 0.047) and RBANS immediate memory subsection scores (control: 91.2, intervention: 102.5, *p* = 0.049) differed significantly between the control and intervention groups. After controlling for MoCA scores, however, the two groups were no longer different in terms of RBANS immediate memory scores at baseline (control: 93.8, intervention: 100.2, *p* = 0.42). Thus, all comparisons between the control and intervention groups were adjusted for MoCA scores.

Pairwise comparisons of total RBANS scores revealed a trend towards a statistically significant decrease from pre-intervention to post-intervention in the control group (99.4 to 93.5, *p* = 0.05) but not in the intervention group (98.1 to 96.0, *p* = 0.65) (Table 3). The RBANS delayed memory subscale score decreased significantly from pre-intervention to post-intervention within the control group but not significantly within the intervention group. Specifically, within the control group, the mean RBANS delayed memory subscale score significantly decreased from 99.7 pre-intervention to 90.9 post-intervention (*p* = 0.010) yet only from 94.5 to 90.2 within the intervention group (*p* = 0.33). While not statistically significant, the RBANS language subscale score had a negative trend from pre-intervention to post-intervention in the control group (96.3 to 91.5, *p* = 0.06) but not in the intervention group (94.8 to 96.7, *p* = 0.95).

Pairwise comparisons of PDQ-39 scores revealed that the cognition subsection score increased significantly (denoting worsening quality of life) from pre-intervention to post-intervention within the control group but not significantly within the intervention group. Specifically, within the control group, the mean PDQ-39 cognition subscale score significantly increased from 23.0 pre-intervention to 32.3 post-intervention (*p* = 0.011) yet only from 24.6 to 26.8 within the intervention group (*p* = 0.99).

When analyzing BrainHQ mean score percentiles (as compared to performance scores for all BrainHQ commercial database users, not adjusted for age) from pre-intervention to post-intervention, the intervention subjects had statistically significant increases in their percentiles across all subdomains (attention, brain speed, and memory) and in their overall BrainHQ scores (Table 4).

## 4. Discussion

This randomized controlled trial was conducted to evaluate the feasibility and impact of a multi-domain, remotely-administered CRT program for PD patients. The results of the study indicate that such a program is feasible, enjoyable, and well-tolerated and may aid in slowing the progression of cognitive decline. Retention was high, with just one withdrawal from the intervention group due to unrelated medical issues. Subjects demonstrated complete adherence to the program across 20 hours of training within 10 weeks. This represents a key strength of the study, as remote administration allowed subjects to make up the few live sessions they missed. Therapist oversight and facilitated group discussion are further strengths of this study. In addition, reported effects of technical difficulties were minimal due to real-time support. Intervention group subjects overwhelmingly reported that they enjoyed both the training and group discussion and would recommend the program to a friend with PD.

The results of neuropsychological evaluation over the course of the study demonstrate more positive trends in the intervention group when compared to the control. We observed a pronounced decrease in total RBANS scores in the control group over time, which trended towards statistical significance. However, a decline of this magnitude was not seen in the intervention group. When comparing RBANS subsection results, the control group also experienced a statistically significant decline in delayed memory not seen in the intervention group. These findings suggest a possible protective effect of CRT on cognition (specifically memory) in the intervention group. The intervention group as a whole also saw statistically significant improvements in attention, brain speed, and memory when comparing performance on exercises at the end of the trial to the baseline. Although this outcome may be confounded by practice effects, it demonstrates that subjects were engaged and their performance improved over time.

The positive trends seen in this study are generally consistent with meta-analyses of comparable trials, which include several examples of remotely-administered programs, and are in consensus that CRT maintains or improves cognition in PD patients [4, 12, 17]. Although the overall findings of neuropsychological evaluation did not achieve statistical significance, this aligns with the results of a 2020 Cochrane review, which did not find consistent evidence of meaningful cognitive benefits for PD patients in cognitive training programs [29]. A recently published randomized controlled trial of PD patients after eight weeks of computerized cognitive training, likewise, found no improvement in overall cognitive function [30]. It is noteworthy, however, that many of the studies reviewed in the meta-analyses described here did not enroll patients with MCI.

There was no significant change in quality of life measures between the intervention or control groups over time, suggesting that overall quality of life was maintained in both groups over the course of the study. The control group, however, did see a statistically significant increase in the PDQ-39 subsection reporting on cognition. This change was not seen in the intervention group and represents another potential positive impact of CRT on this domain. These findings align with other studies and most meta-analyses on the subject, which have not consistently identified improvements in noncognitive factors such as depression and patient quality of life [4, 12, 13, 17, 29, 31]. Combining remote CRT with complementary non-pharmacological interventions, such as active theatre therapy (which has been shown to improve psychological well-being in PD but not cognition), could address this deficit and make for a more comprehensive approach to treatment [32]. Notably, nearly three-quarters of the intervention group reported subjective improvements in their daily lives, specifically in memory and attention. However, these observations were not reflected as significant improvements over time on RBANS or the PDQ-39.

### 4.1. Advantages

Remotely-administered CRT has several advantages, both for PD patients as a cohort and more broadly in the context of the COVID-19 pandemic. As PD progresses, many patients struggle with ambulation and lose the ability to drive, creating barriers to in-person therapy and a need for more accessible options. With the onset of the COVID-19 pandemic, this need was compounded and demand for interventions that could be conducted without placing the patient at additional risk arose, as seen in our previous work [33, 34]. Home-based programs such as ours are beneficial for PD patients with limited mobility, as well as for rural patients and those with comorbid conditions requiring isolation. CRT also has a positive safety profile and, consistent with prior literature [12], remote CRT was not associated with any identified adverse events over the course of this study.

### 4.2. Limitations

While our findings established the feasibility of this remote CRT intervention, there were several limitations in the design of the study, and its impact on cognition should be interpreted accordingly. For one, it did not include an active control group, such as sham cognitive training. Such training was only employed in three out of 17 studies included in a recent meta-analysis in which five others used either a passive control or physical exercise routine shared between groups, as reported here [17]. Our study intentionally omitted a truly passive control group, one that did not exercise, as the comparison of subjects who exercise to those who do not could introduce confounding variables (given the known benefits of physical exercise in PD) [24]. Additionally, given the nature of the intervention and size of the research team, neither the participants nor the investigators were blinded to the subjects' group assignments. The small sample size and homogenous demographics of the subject pool (the targeted PD exercise program from which participants were recruited) could also be seen as limitations to this work. Demographically, participants in this study were predominantly White, highly-educated, and physically active at baseline. Three subjects also withdrew from the study; however, the resulting attrition rate of 12.5% was below our anticipated 15%, suggesting that the study was still adequately powered. There were also a few instances of missing data points from PDQ-39 surveys. Although GEE analysis was applied to best handle the missing data, this study might have been subject to some degree of attrition bias.

Given current literature findings, which indicate that CRT has some cognitive benefit for PD patients, it could be considered whether such training simply has lower utility as a supplement to physical exercise. If there is a hypothetical maximum cognitive benefit one can attain from non-pharmacological interventions, a ceiling effect is possible, in which subjects already benefiting cognitively from a targeted physical exercise program can only benefit so much from the addition of CRT. Other studies have touted the benefits of such a combined approach compared to either program alone and would dispute this claim [25, 26]. However, both studies enrolled only PD patients with MCI, complicating comparison to the present study, and it is worth considering whether such non-pharmacological interventions might play a role in preventing the development of MCI.

Participants in this study had a minimum MoCA score of 20, meaning both cognitively normal scores (≥26) and some scores within the range of MCI (18–25) were included [35]. This cut-off was set to ensure that subjects could understand the exercises and is an established screen for Parkinson's disease dementia (PDD) [36], as it is unclear whether CRT is efficacious following PDD onset [17, 29, 37]. Of the subjects who completed the trial, eleven (52.4%) scored a 26 or above at the baseline. However, a disproportionate number of these subjects (*n* = 7; 63.6%) were randomly assigned to the intervention group. This explains the statistically significant difference in MoCA scores at baseline between groups. Although outcome data were adjusted to control for this, it resulted in an intervention cohort who performed higher on neuropsychological testing at baseline than the control group. If we consider the compensation hypothesis for CRT, as has been suggested previously [21], it is possible that the intervention group had less room for improvement, contextualizing our findings. However, prior studies have variably shown support for both the compensation hypothesis and the magnification hypothesis, and such a conclusion cannot be drawn from this study [19]. Regardless, it is possible that some members of the control group were on a more severe, deteriorating course, and this should be considered in the interpretation of our results beyond feasibility.

Lastly, this study only evaluated subjects at two timepoints: baseline and immediately post-intervention. It is possible that a longer follow-up period was required for significant group differences to develop or that continued remote training might have brought about improvements not detected immediately following the intervention. At least two prior cognitive training trials with PD patients reported sustained cognitive performance in their intervention groups at 12–18 months follow-up [38, 39], although both saw significant differences relative to control at post-test, meaning comparable longitudinal results for this study are unlikely. Another such trial, however, focused on working memory and found evidence of improvement only after a three-month follow-up, with no identifiable effects in the short term [21]. Further evaluation of this cohort would be necessary to determine whether such a delayed cognitive protective effect was a benefit experienced by the intervention group.

## 5. Conclusion

We have demonstrated that remotely-administered CRT for patients with PD is logistically possible and well-tolerated by participants already enrolled in a targeted exercise program. To determine the feasibility and impact of such a combined program, a randomized controlled trial was conducted in which a cohort of PD patients participated in 20 hour-long supervised CRT sessions over the course of 10 weeks. Adherence was high, and subjects overwhelmingly enjoyed both the cognitive exercises and group discussion. Analysis of the results of neuropsychological evaluation and quality of life measures helped identify that remote CRT was effective in preventing the declines in objective delayed memory and subjective cognition seen in the control group. However, given the limitations of our study design, larger randomized controlled trials are needed to verify the nonsignificant decline in total RBANS scores seen only in the control group and to support the claims of subjective cognitive improvements made by the intervention group. Further investigations could determine whether such a program is cognitively protective longitudinally.

## Figures and Tables

**Table 1:** Subject demographics.

Characteristic	Control	Intervention	*p* value
Number of subjects	10	11	-
Age in years, mean (SD)	68.2 (10.2)	69.1 (6.3)	0.81
Male sex, no. (%)	6 (60.0%)	6 (54.5%)	0.80
White race, no. (%)	10 (100.0%)	10 (90.9%)	0.33
Non-Hispanic ethnicity, no. (%)	10 (100.0%)	11 (100.0%)	1.00

Highest level of education completed, no. (%)			
High school	0 (0.0%)	1 (9.1%)	0.42
College	6 (60.0%)	4 (36.4%)	
Graduate school	4 (40.0%)	6 (54.5%)	
Currently employed, no. (%)	1 (10.0%)	2 (18.2%)	0.66

**Table 2:** Intervention group quotes recorded during group discussion.

Domain	Theme	Supporting quotes
Cognition	Memory	*“I've noticed I'm * ***remembering *** *to lock the door more often”*
		*“(My wife) thought my * ***recall of history *** *was improving. Names, things like that”*
		*“I noticed how when… they text you a code, my ability to * ***recall *** (***the numbers****) without writing them down has improved significantly”*
	Thought process	*“I've found that it makes me think in a different way. It kind of * ***broadened my mind *** *and made me a bit more observant than I was before”*
		*“(The exercises) * ***organize me *** *… Yesterday I went shopping and I put things in alphabetical order in my head”*
		*“I used to get so upset about things and I just think that maybe these games are making me think a little differently. We're all thinking a little outside the box… and you * ***learn different ways of handling situations*** *”*
	Attention	*“I have noticed that I am * ***more focused *** *at work. For example, not as distracted by staff chatter, phones ringing, music, etc”*
		*“I'm more focused for sure. Paying attention better… My wife notices I'm * ***less distracted*** *”*
		*“I'm not as distracted as I was in the past, I can focus better, * ***I can block things out*** *”*

Emotional well-being	Self-confidence	*“I found myself * ***more confident *** *in communicating with other people”*
		*“I feel increased * ***self-efficacy*** *”*
		*“As I keep going I seem to be * ***more positive about myself *** *rather than beating myself down”*
	Patience	*“I think all these exercises are actually making me * ***more patient*** *”*
		*“I'm feeling like I'm getting more patient about things in my own life. Instead of getting anxious * ***I'm able to calm myself down *** *…”*
		*“Oh, this program has been a godsend, I'm so thankful for it. I feel like it has made me a better person. I'm more patient… I've even * ***used some of our strategies for the games to help (my husband) *** *get dressed in the morning”*
	Mood	*“I am always in a * ***good mood *** *... It gives me a high afterwards, the same feeling I get after physical exercise”*
		*“It * ***relaxes *** *me. You have to focus and I am less stressed because I am able to get this other stuff I have going on in my life off of my mind”*
		*“Feels like going to the gym for my brain, I feel * ***more alert*** *”*

Activities of daily living	Language	*“My wife says I'm asking her to * ***repeat herself less often*** *”*
		*“I think I'm searching for words less often… * ***words come to me easier *** *in conversation”*
		*“I noticed that my * ***ability to read improved*** *. Prior to the study I was reading about one or two books a month. During the study I was easily reading a book a week”*
	Visuospatial awareness	*“I think I'm more * ***aware of my surroundings*** *. I have cats, and maybe it's my peripheral vision, but they'll run between my legs and I've been tripping over them less”*

**Table 3:** Comparison of mean outcome measure score changes between intervention and control groups.

Variable	*p* value	Intervention (*n* = 11)		*p* value	Control (*n* = 10)		*p* value	*p* value
	Pre/Pre	Pre-session	Post-session	I Pre/Post	Pre-session	Post-session	C Pre/Post	Post/Post
*RBANS*								
Total	0.71	98.1 (89.8, 106.4)	96.0 (87.5, 104.5)	0.65	99.4 (90.6, 108.1)	93.5 (84.6, 102.4)	0.05	0.65
Immediate memory	0.42	100.2 (93.8, 106.7)	95.5 (89.0, 102.0)	0.35	93.8 (87.0, 100.6)	93.4 (86.6, 100.3)	0.95	0.95
Visuospatial/construction	0.99	101.2 (89.9, 112.4)	104.0 (91.4, 116.5)	0.96	109.0 (97.2,120.9)	106.9 (93.8, 120.1)	0.98	0.99
Language	0.95	94.8 (85.1, 104.5)	96.7 (89.4, 104.1)	0.95	96.3 (86.1, 106.6)	91.5 (83.8, 99.3)	0.06	0.46
Attention	0.84	101.2 (91.4, 110.9)	97.8 (87.5, 108.1)	0.58	100.5 (90.3, 110.8)	95.2 (84.3, 106.0)	0.79	0.84
Delayed memory	0.88	94.5 (85.4, 103.6)	90.2 (80.8, 99.5)	0.33	**99.7 (90.0, 109.3)**	**90.9 (81.1, 100.7)**	**0.010**	0.88

*PDQ-39*								
Total	0.99	18.5 (10.1, 26.8)	19.0 (10.7, 27.4)	0.99	18.4 (9.6, 27.2)	22.1 (13.4, 30.9)	0.22	0.88
Mobility	0.99	17.0 (7.0, 27.0)	17.8 (5.6, 29.9)	0.99	14.6 (4.0, 25.1)	17.2 (4.4, 30.0)	0.53	0.99
ADL	0.78	11.0 (1.5, 20.5)	14.1 (3.9, 24.3)	0.41	18.7 (8.7, 28.7)	19.1 (8.3, 29.8)	0.75	0.79
Emotional well-being	0.88	21.6 (9.3, 33.8)	20.1 (9.2, 31.0)	0.88	23.2 (10.3, 36.1)	27.3 (15.8, 38.7)	0.76	0.76
Stigma	0.97	12.9 (0.0, 26.2)	16.1 (0.0, 32.8)	0.97	22.7 (8.6, 36.7)	26.1 (8.4, 43.7)	0.78	0.94
Social support	0.43	16.0 (6.6, 25.4)	11.4 (2.2, 20.6)	0.57	4.9 (0.0, 14.8)	11.7 (2.0, 21.3)	0.56	0.99
Cognition	0.99	24.6 (13.0, 36.1)	26.8 (15.1, 38.6)	0.99	**23.0 (10.8, 35.1)**	**32.3 (20.0, 44.7)**	**0.011**	0.57
Communication	0.96	12.4 (0.1, 24.6)	15.0 (3.3, 26.7)	0.83	11.4 (0.0, 24.3)	14.3 (2.0, 26.7)	0.96	0.96
Bodily discomfort	0.95	32.5 (19.6, 45.5)	31.0 (18.2, 43.8)	0.95	28.4 (14.8, 42.0)	29.3 (15.8, 42.8)	0.96	0.96

Reported are the MoCA-adjusted means (95% CI). Reported *p* values are the results of running generalized estimating equation for the pairwise comparisons among pre-intervention, post-intervention, pre-control, and post-control, controlling for MoCA, with the sequential Sidak method for multiplicity correction. pre/pre, pre-intervention vs. pre-control (baseline). I pre/post, pre-intervention vs. post-intervention. C pre/post, pre-control vs. post-control. post/post, post-intervention vs. post-control. ADL, activities of daily living.

**Table 4:** Comparison of mean BrainHQ outcome score changes within the intervention group.

Variable	Intervention (*n* = 11)		*p* value^*∗*^
	Pre-session	Post-session	
BrainHQ percentiles, mean (SD)			
Overall	**37.8 (13.4)**	**53.9 (15.4)**	**0.002**
Attention	**17.0 (14.5)**	**32.2 (19.5)**	**0.003**
Brain speed	**38.0 (14.5)**	**54.3 (16.3)**	**0.002**
Memory	**45.3 (15.4)**	**61.5 (16.6)**	**0.002**

^*∗*^ *p* values are the result of running the related-sample Wilcoxon signed rank test.

## Data Availability

The data generated in this study can be obtained from the corresponding author upon request.
